# Long-Term Outcomes of Mechanical Versus Bioprosthetic Aortic Valve Replacement: A Systematic Review and Meta-Analysis

**DOI:** 10.7759/cureus.52550

**Published:** 2024-01-19

**Authors:** Dilip K Vankayalapati, Omotayo Segun-Omosehin, Nour El Ghazal, Rohan Suresh Daniel, Joe El Haddad, Rania Mansour, Nathanael Yap, Shahid Miangul, Hayato Nakanishi, Christian A Than

**Affiliations:** 1 General Surgery, Buckinghamshire Healthcare NHS Trust, Buckinghamshire, GBR; 2 Cardiothoracic Surgery, St George's University of London, London, GBR; 3 Cardiothoracic Surgery, University of Nicosia Medical School, Nicosia, CYP; 4 Biomedical Sciences, The University of Queensland, Brisbane, AUS

**Keywords:** survival, bioprosthetic valve, mechanical valve, valve replacement, aortic stenosis

## Abstract

This study aimed to investigate the safety and efficacy of bioprosthetic (BV) versus mechanical valves (MV) on long-term outcomes in 50- to 70-year-old aortic stenosis (AS) patients. A literature search for articles published until April 2023 yielded 13 eligible studies, with 15,320 patients divided into BV (n = 7,320) and MV (n = 8,000) cohorts. The review was registered prospectively with PROSPERO (CRD42021278777). MV demonstrated a favorable hazard ratio (HR: 1.12, 95% CI: 1.00-1.25, I^2 ^= 60%) and higher survival rates at 5 (OR:1.13, 95% CI: 1.02-1.25, I^2 ^= 42%) and 10 years (OR: 1.13, 95% CI: 1.05-1.23, I^2 ^= 0%). At 15 years, stroke incidence was comparable (OR: 1.12, 95% CI: 0.98-1.27, I^2 ^= 4%). BV showed lower bleeding events (OR: 1.7, 95% CI: 1.18-2.46, I^2 ^= 88%), but MV replacement showed lower reoperation incidence (OR: 0.27, 95% CI: 0.18-0.42, I^2 ^= 85%). MV appears favorable for the long-term approach in AS management compared to BV.

## Introduction and background

Aortic stenosis (AS) is a common valvular pathology in the United States, with a markedly higher prevalence of 5% in populations aged 65 years and older [1]. Though no medical therapies exist to prevent disease progression, aortic valve replacement (AVR) serves as the definitive management for symptomatic and severe AS [2-4]. Clinical evidence has demonstrated improved symptomatic management, quality of life, and promising outcomes for long-term mortality [5-7]. As surgical AVR continues to serve as the gold standard treatment for patients with severe AS, the choice of a mechanical valve (MV) or bioprosthetic valve (BV) is subject to various factors such as age, indications for anticoagulation therapy, risks of valve re-intervention and patient values and preferences [4].

Though noted for their durability, MVs require the use of lifelong anticoagulation treatment due to the high thrombogenicity of their constituent materials to avoid complications from clot formation [8,9]. The BVs introduced in the 1960s expanded the options for valve prosthetics and overcame the coagulation challenge posed by MVs [8,9]. However, BVs carry a higher risk of structural valve deterioration (SVD) and subsequent reoperations for valvular replacement when compared to MVs [8]. The average lifespan of BVs is approximately 15 years with a higher risk of SVD in younger patients due to a pronounced immunologic response [8]. With the risks-to-benefits ratio in mind, the American Heart Association/American College of Cardiology (AHA/ACC) recommends the use of MVs in patients under 50 years of age [4]. Similarly, the European Society of Cardiology/European Association of Cardiothoracic Surgery (ESC/EACTS) recommends MVs in patients under 60 and BVs for patients over 65 [10].

While clear recommendations exist by the preceding associations for patients under 50 and over 65 years of age, the choice of valvular type remains up to the clinician and patient discretion between these age ranges [4]. To provide optimal patient treatment, both short- and long-term outcomes must be considered in the decision-making process for each case. Evaluation of the short-term and mid-term survival rates between BV and MV at various age ranges has been previously investigated [11]. However, to our knowledge, no meta-analyses of existing studies have compared outcomes of BV and MV for stroke, bleeding, and reoperation rates at 5-, 10-, and 15-years postoperation for long-term elucidation. Therefore, this meta-analysis aims to assess the long-term survival and clinical outcomes in recipients of biological versus mechanical AVR between the ages of 50 and 70 years old. Clarification of such clinical outcomes will serve as a foundation upon which clinicians and patients may build for shared decision-making on treatment.

## Review

Methods

Data Sources and Search Strategies

A comprehensive search of several databases from inception to April 20, 2023, was conducted in compliance with the Preferred Reporting Items for Systematic Reviews and Meta-analyses (PRISMA) guidelines [12]. The databases included Ovid MEDLINE(R) and Epub Ahead of Print, In-Process and Other Non-Indexed Citations and Daily, Ovid Embase, Ovid Cochrane Central Register of Controlled Trials, Ovid Cochrane Database of Systematic Reviews, and Scopus. The search strategy was designed and conducted by an experienced librarian with input from the study’s principal investigator. Controlled vocabulary supplemented with keywords was used to search for studies describing AVR with bioprosthetic or mechanical valves in patients 50-70 years old. The review was registered prospectively with PROSPERO (CRD42021278777). The actual strategy listing all search terms used and how they are combined is available in Table 1.

**Table 1:** Search strategy

Database	Searches
Ovid Medline	Aortic Valve/((aorta* or aortic*) adj2 valve*).ti,ab,hw,kw. 1 or 2 Bioprosthesis/(((biologic* or tissue*) adj3 (implant* or prosthesis or prostheses or prosthetic)) or (bio-prosthe* or bioprosthe*)).ti,ab,hw,kw. 4 or 5 3 and 6 (mechanical and (implant* or prosthesis or prostheses or prosthetic or valve)).ti,ab,kw. (3 and 8) or mechanical aortic valve prosthesis/7 and 9 ((Heart Valve Prosthesis Implantation/and ((biolog* or bioprosth* or tissue*) and mechanical and (aorta* or aortic*)).ti,ab,hw,kw.) or exp aortic valve replacement/) and ((biolog* or bioprosth* or tissue*) and mechanical).ti,ab,hw,kw. 10 or 11 ("middle age*" or "middle-age*").ti,ab,hw,kw. (50* or 51* or 52* or 53* or 54* or 55* or 56* or 57* or 58* or 59* or 60* or 61* or 62* or 63* or 64* or 65* or 66* or 67* or 68* or 69* or 70*).ti,ab. limit 14 to ("middle age (45 to 64 years)" or "middle aged (45 plus years)") (Limit not valid in CCTR,CDSR,Embase; records were retained] 13 or 14 or 15 12 and 16 (conference abstract or conference review or editorial or erratum or note or addresses or autobiography or bibliography or biography or blogs or comment or dictionary or directory or interactive tutorial or interview or lectures or legal cases or legislation or news or newspaper article or patient education handout or periodical index or portraits or published erratum or video-audio media or webcasts).mp. or conference abstract.st. 17 not 18 Limit 19 to yr=”2021 -Current”
Embase	'aortic valve'/exp ((aorta* OR aortic*) NEAR/2 valve*):ti,ab,kw #1 OR #2 'bioprosthesis'/exp (((biologic* OR tissue*) NEAR/3 (implant* OR prosthesis OR prostheses OR prosthetic)):ti,ab,kw) OR 'bio prosthe*':ti,ab,kw OR bioprosthe*:ti,ab,kw #4 OR #5 #3 AND #6 mechanical:ti,ab,kw AND (implant*:ti,ab,kw OR prosthesis:ti,ab,kw OR prostheses:ti,ab,kw OR prosthetic:ti,ab,kw OR valve:ti,ab,kw) #3 AND #8 'mechanical aortic valve prosthesis'/exp #7 AND #10 'heart valve prosthesis implantation'/exp (biolog*:ti,ab,kw OR bioprosth*:ti,ab,kw OR tissue*:ti,ab,kw) AND mechanical:ti,ab,kw AND (aorta*:ti,ab,kw OR aortic*:ti,ab,kw) #12 AND #13 ‘aortic valve replacement'/exp OR 'aortic valve replacement' #14 OR #15 (biolog*:ti,ab,kw OR bioprosth*:ti,ab,kw OR tissue*:ti,ab,kw) AND mechanical:ti,ab,kw #16 AND #17 #11 OR #18 'middle age*':ti,ab,kw OR 'middle-age*':ti,ab,kw #19 AND #22 #20 OR #21 62*:ti,ab OR 63*:ti,ab OR 64*:ti,ab OR 65*:ti,ab OR 66*:ti,ab OR 67*:ti,ab OR 68*:ti,ab OR 69*:ti,ab OR 70*:ti,ab 50*:ti,ab OR 51*:ti,ab OR 52*:ti,ab OR 53*:ti,ab OR 54*:ti,ab OR 55*:ti,ab OR 56*:ti,ab OR 57*:ti,ab OR 58*:ti,ab OR 59*:ti,ab OR 60*:ti,ab OR 61*:ti,ab OR #23 AND ('Conference Abstract'/it OR 'Editorial'/it OR 'Note'/it) #23 NOT #24 #23 NOT #24 AND (09-09-2021)/sd NOT (01-01-2024)/sd
Cochrane	MeSH descriptor: (Aortic Valve) explode all trees (((aorta* or aortic*) NEAR/2 valve*)):ti,ab,kw (Word variations have been searched) #1 OR #2 MeSH descriptor: (Bioprosthesis) explode all trees ((((biologic* or tissue*) NEAR/3 (implant* or prosthesis or prostheses or prosthetic)) or (bio-prosthe* or bioprosthe*))):ti,ab,kw (Word variations have been searched) #4 OR #5 #3 AND #6 ((mechanical and (implant* or prosthesis or prostheses or prosthetic or valve))):ti,ab,kw (Word variations have been searched) #3 AND #8 #7 AND #9 MeSH descriptor: (Heart Valve Prosthesis Implantation) explode all trees ((biolog* OR bioprosth* OR tissue*) AND mechanical AND (aorta* OR aortic*)):ti,ab,kw (Word variations have been searched) #11 AND #12 (aortic valve replacement):ti,ab,kw (Word variations have been searched) ((biolog* or bioprosth* or tissue*) and mechanical):ti,ab,kw (Word variations have been searched) #15 AND #14 #13 OR #14 #10 OR #17 (("middle age*" or "middle-age*")):ti,ab,kw (Word variations have been searched) ((50* or 51* or 52* or 53* or 54* or 55* or 56* or 57* or 58* or 59* or 60* or 61* or 62* or 63* or 64* or 65* or 66* or 67* or 68* or 69* or 70*)):ti (Word variations have been searched) ((50* or 51* or 52* or 53* or 54* or 55* or 56* or 57* or 58* or 59* or 60* or 61* or 62* or 63* or 64* or 65* or 66* or 67* or 68* or 69* or 70*)):ab (Word variations have been searched) #20 OR #21 #19 OR #22 #18 AND #23
Scopus	TITLE-ABS-KEY (aorta OR aortic) TITLE-ABS-KEY (((biologic* or tissue*) W/3 (implant* or prosthesis or prostheses or prosthetic)) or (bio-prosthe* or bioprosthe*)) TITLE-ABS-KEY (mechanical and (implant* or prosthesis or prostheses or prosthetic or valve)) 1 and 2 and 3 INDEX(embase) OR INDEX(medline) OR PMID(0* OR 1* OR 2* OR 3* OR 4* OR 5* OR 6* OR 7* OR 8* OR 9*) 4 not 5 DOCTYPE(ed) OR DOCTYPE(bk) OR DOCTYPE(er) OR DOCTYPE(no) OR DOCTYPE(sh) OR DOCTYPE(ch) 6 not 7 8 AND (LIMIT-TO (PUBYEAR, 2023) OR LIMIT-TO (PUBYEAR, 2022) OR LIMIT-TO (PUBYEAR, 2021))

Eligibility Criteria and Quality Assessment

Eligible studies were all propensity-matched retrospective studies (PSM) or randomized controlled trials (RCTs) that must meet all of the following inclusion criteria: 1) Comparative studies of adult participants 50 to 70 years of age who underwent AVR with either a mechanical or BV; 2) Outcomes of overall survival rate or at least one of the secondary outcomes of stroke, major bleeding or reoperation rates; 3) Minimum of a five years follow up for all reported outcomes. Single-arm treatment studies and literature with overlap between authors, centers, or patient cohorts evaluated in published literature were excluded from the study. Case reports, case series, conference abstracts and/or abstracts, and articles that were not reported in English were also excluded. The quality of each study was independently evaluated by two authors (DKV and OSO) using the Newcastle-Ottawa Scale [13].

Data Extraction

Survival rates and secondary outcomes at 1, 2, 3, 4, 5, 7, 10, 12, and 15 years for each valve arm were extracted and calculated from either reported literature values or raw data. When survival rates could not be directly obtained from the publication, Kaplan-Meier (KM) curves were digitized using WebPlotDigitizer version 4.4 (https://automeris.io/WebPlotDigitizer/) and iteratively computed to generate individual patient data and survival rates, using the algorithm from Guyot and colleagues [14]. Furthermore, the hazard ratio (HR) with a 95% confidence interval was obtained based on the raw data obtained from KM curves in the previous step and extracted to calculate logHR and standard error as explained by Guyot and colleagues [14]. These analyses were completed using R Studio software (version 1.4.1106, The R Foundation for Statistical Computing, Boston, United States).

Statistical Analysis

The pooled means and proportions of our data were analyzed using an inverse variance method for continuous data and the Mantel-Haenszel method for dichotomous data, which assigns the weight of each study based on its variance. The pooled estimate of HR was analyzed using an inverse-variance-weighted average of the individual studies [15]. A direct comparison between the two techniques was conducted by assessing studies that reported outcomes of both treatments (two-arm analysis). The heterogeneity of effect size estimates across the studies was quantified using the Q statistic and I^2^ (p < 0.10 was considered significant). An I^2^ value of 0-25% indicates insignificant statistical heterogeneity, 26-50% low heterogeneity, and 51-100% high heterogeneity [16]. The Random-effects model was used when the value of I^2^ was >50% and the fixed-effects model was used for I^2^ < 50%. Publication bias was assessed using a funnel plot [17]. If mean and standard deviation (SD) were not available, the median was converted to mean using the formulas from the Cochrane Handbook for Systematic Reviews of Interventions [15]. Data analysis was performed using RevMan software (version 5.4, Cochrane Collaboration, London, United Kingdom).

Results

Study Selection and Patient Characteristics

**Figure 1:**
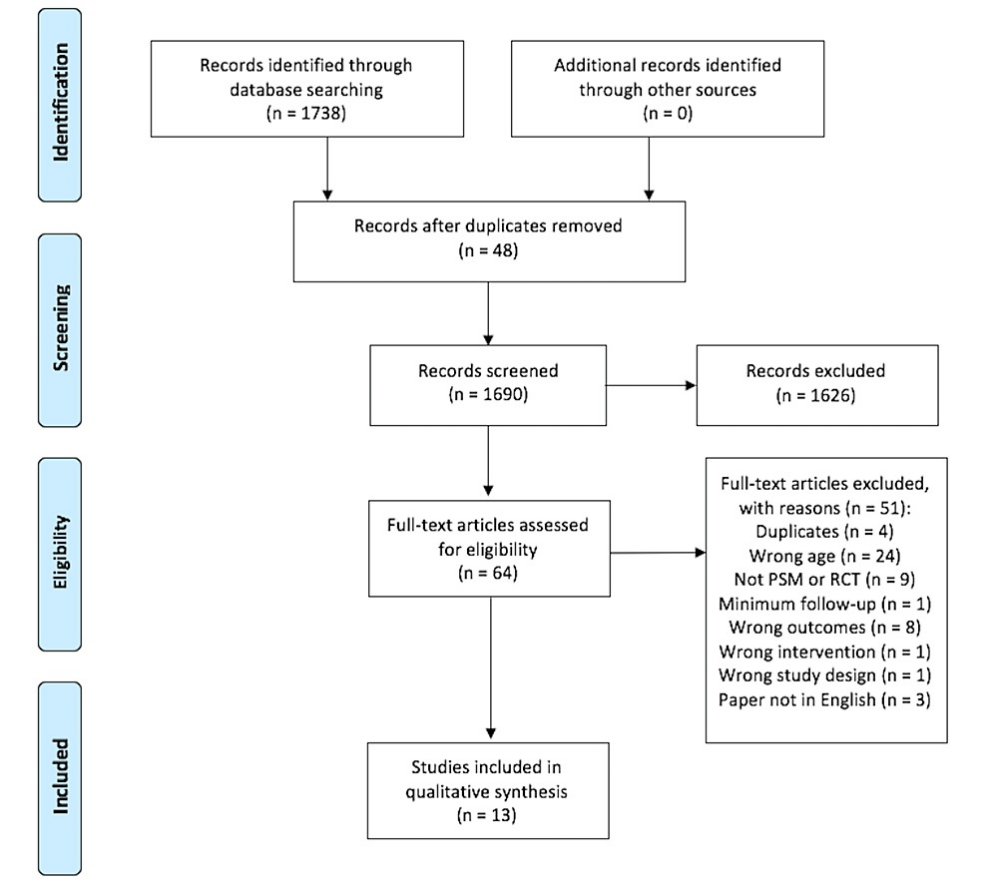
PRISMA flow diagram PRISMA: Preferred Reporting Items for Systematic Reviews and Meta-Analyses

The initial literature search yielded 1,737 studies. After removing duplicates, the articles were screened for inclusion and exclusion criteria, and 64 studies were retained for full-text review. Thirteen studies involving 15,320 patients were included in this meta-analysis. Of the 13 studies, one was an RCT [18], and the remaining were PSM studies [19-30]. PRISMA flowchart of the study selection process is depicted in Figure 1.

The mean follow-up period for combined MV and BV cohorts ranged from 64.8 to 132 months. St. Jude’s’ medical valve was used in the MV cohort of six included studies [18,20,22,23,29,30]. The Carpentier-Edwards valve was reported in the BV cohort of four included studies [18,20,29,30]. The baseline characteristics of the included studies are described in Table 2.

**Table 2:** Baseline characteristics of included studies BV: Bioprosthetic valve; MV: Mechanical valve; n: Number of participants; NR: Not reported; PSM: propensity-matched; RCT: Randomized control trial; SD: Standard deviation; USA: United States of America * Standard deviation was not reported

Author	Year	Country	Number of participants (n)	Number of male participants (n)	Mean age (years) ± SD	Mechanical valve	Biological valve	MV mean follow-up (months) ± SD	BV mean follow-up (months) ± SD
Stassano et al. [18]	2009	Italy	310	144	63.8 ± 6.0	St Jude Medical; CarboMedics	Carpentier-Edwards SAV; Carpentier-Edwards Pericardial	106.0 ± 28.0	106.0 ± 28.0
Chiang et al. [19]	2014	USA	2002	1279	61.5 ± 5.5	NR	NR	130.8 ± 50.7	127.2 ± 50.7
Rodríguez-Caulo et al. [20]	2021	Spain	2733	1839	60.8 ± 4.0	CarboMedics; St Jude Medical Regent; Sorin Bicarbon; ATS Open Pivot; On-X	Carpentier-Edwards; Mitroflow; Mosaic; Trifecta	102.0 ± 57.6	87.6 ± 57.6
Glaser et al. [21]	2015	Sweden	2198	1466	62.2 ± 4.8	NR	NR	80.4 ± 50.4	78.0 ± 46.8
Attia et al. [22]	2021	USA	1054	663	54.0 ± 13.0	St Jude devices; Carbomedics	Perimount Bovine pericardial	NR	NR
Vogt et al. [23]	2022	Germany	1220	868	58.2 ± 4.5	ATS/Open Pivot; St Jude Medical Masters; St Jude Medical Regent; Carbomedics	Perimount	NR	NR
Kim et al. [24]	2022	Korea	2858	1807	63.0 ± 4.3	NR	NR	NR	NR
Hu et al. [25]	2021	China	692	431	62.5 ± 5.3	NR	NR	76.8 ± 56.0	80.4 ± 59.6
Huckaby et al. [26]	2020	USA	392	132	61.5 ± 6.3	NR	NR	NR	NR
Iribarne et al. [27]	2019	USA	1449	1382	58.5 ± 0.2	NR	NR	141.6 ± 244.4	75.6 ± 238.9
Alex et al. [28]	2017	Canada	236	163	61.8*	NR	NR	102.0 ± 75.6	69.6 ± 54.2
Sakamoto et al. [29]	2016	Japan	56	28	64.8 ± 2.7	St Jude Medical Standard; St Jude Medical Regent; ATS; CarboMedics	Carpentier-Edwards Perimount; Carpentier-Edwards Magna; Carpentier-Edwards Magna Ease; Medtronic Mosaic; Medtronic Ultra; St Jude Medical Trifecta; St Jude Medical Epic	64.8 ± 54.0	64.8 ± 54.0
Roumieh et al. [30]	2014	Germany	120	86	61.3 ± 3.0	St Jude Medical	Medtronic Mosaic; Sorin Mitroflow; Carpentier-Edwards Perimount; St Jude Medical Toronto SPV	128.0 ± 54.0	106.0 ± 46.0

A total of 8,000 patients were included in the MV cohort while 7,320 patients were included in the BV cohort. Of these groups, 5,325 (66.5%) and 4,965 (67.8%) were male in the MV and BV groups respectively. The clinical characteristics of the included studies are reported in Table 3.

**Table 3:** Cumulative clinical characteristics of included studies AF: Atrial fibrillation; BV: Bioprosthetic valve; CABG: Coronary artery bypass graft; CAD: Coronary artery disease; COPD: Chronic obstructive pulmonary disease; CKD: Chronic kidney disease; MI: Myocardial infarction; MV: Mechanical valve; n: Sample size; N: total number of patients included across studies; PCI: Percutaneous coronary intervention; PVD: Peripheral vascular disease; SD: Standard deviation

Patient characteristics	Mechanical valve (n = 8000)	Bioprosthetic valve (n = 7320)
Demographic characteristics		
Age, mean ± SD	61.0 ± 5.0	61.1 ± 4.9
Male, n (%)	5325 (66.6)	4965 (67.8)
Female, n (%)	2675 (33.4)	2355 (32.2)
Comorbidities		
Hypertension, n/N (%)	3088/7148 (43.2)	2494/6237 (40.0)
Hyperlipidemia, n/N (%)	1075/3039 (35.4)	627/2128 (29.5)
Smoking, n/N (%)	475/2074 (22.9)	491/2074 (23.7)
Diabetes, n/N (%)	1554/7940 (19.6)	1327/7260 (18.3)
Heart failure, n/N (%)	692/4452 (15.5)	694/4683 (14.8)
CAD, n/N (%)	517/3804 (13.6)	537/4035 (13.3)
COPD, n/N (%)	824/7912 (10.4)	702/7232 (9.7)
AF, n/N (%)	452/5523 (8.2)	422/5452 (7.7)
CKD, n/N (%)	311/4781 (6.5)	244/3870 (6.3)
History of cancer, n/N (%)	172/3529 (4.9)	173/3529 (4.9)
Stroke, n/N (%)	296/6684 (4.4)	262/5773 (4.5)
History of MI, n/N (%)	290/6565 (4.4)	247/5885 (4.2)
History of PVD, n/N (%)	343/7940 (4.3)	266/7260 (3.7)
Valvular etiology		
Aortic stenosis, n/N (%)	1635/2149 (76.1)	1600/2149 (74.5)
Aortic insufficiency, n/N (%)	723/1625 (44.5)	700/1625 (43.1)
Other, n/N (%)	327/783 (41.8)	346/783 (44.2)
Degenerative, n/N (%)	189/542 (34.9)	198/542 (36.5)
Rheumatic, n/N (%)	162/542 (29.9)	152/542 (28.0)
Surgical intervention		
Elective, n/N (%)	857/1533 (55.9)	849/1764 (48.1)
Urgent, n/N (%)	575/4661 (12.3)	571/4892 (11.7)
Intraoperative characteristics		
Concomitant CABG, n/N (%)	482/2513 (19.2)	488/2744 (17.8)
PCI, n/N (%)	100/3515 (2.8)	91/3746 (2.4)

The individual clinical characteristics of each included study are reported in Tables 4-6.

**Table 4:** Clinical characteristics (demographics) of each included study BV: Bioprosthetic valve; MV: Mechanical valve; n: Number of participants; SD: Standard deviation * Standard deviations were not reported

Study	Number of participants (n)		Number of male participants (n)		Mean age ± SD	
	MV	BV	MV	BV	MV	BV
Stassano et al., 2009 [18]	155	155	66	78	64.0 ± 7.6	63.5 ± 3.9
Chiang et al., 2014 [19]	1001	1001	645	634	61.5 ± 5.3	61.5 ± 5.7
Rodríguez-Caulo et al., 2021 [20]	1822	911	1229	610	60.8 ± 3.9	60.9 ± 4.1
Glaser et al., 2015 [21]	1099	1099	719	747	62.3 ± 4.5	62.1 ± 5.1
Attia et al., 2021 [22]	527	527	334	329	54.0 ± 12.0	54.0 ± 14.0
Vogt et al., 2022 [23]	610	610	431	437	58.2 ± 4.5	58.2 ± 4.5
Kim et al., 2022 [24]	1429	1429	900	907	62.9 ± 4.3	63.1 ± 4.2
Hu et al., 2021 [25]	346	346	220	211	62.3 ± 5.5	62.7 ± 5.1
Huckaby et al., 2020 [26]	196	196	66	66	61.0 ± 5.9	62.0 ± 6.7
Iribarne et al., 2019 [27]	609	840	576	806	58.4 ± 0.2	58.6 ± 0.2
Alex et al., 2017 [28]	118	118	79	84	62.0*	61.5*
Sakamoto et al., 2016 [29]	28	28	16	12	64.3 ± 2.8	65.3 ± 2.6
Roumieh et al., 2014 [30]	60	60	43	43	61.0 ± 3.0	61.5 ± 3.0

**Table 5:** Clinical characteristics (comorbidities) of each included study AF: Atrial fibrillation; BV: Bioprosthetic valve; CAD: Coronary artery disease; CKD: Chronic kidney disease; COPD: Chronic obstructive pulmonary disease; HF: Heart failure; MI: Myocardial ischemia; MV: Mechanical valve; n: Number of participants; NR: Not reported; PVD: Peripheral vascular disease

Study	Comorbidities (n)													
	Smoking	Hyperlipidemia	Hypertension	Diabetes	COPD	CAD	AF	Stroke	CKD	HF	History of PVD	History of cancer	History of MI	
Stassano et al., 2009 [18]	MV: NR	MV: NR	MV: NR	MV: 23	MV: 43	MV: 41	MV: NR	MV: NR	MV: 10	MV: NR	MV: 21	MV: NR	MV: 8	
	BV: NR	BV: NR	BV: NR	BV: 19	BV: 39	BV: 52	BV: NR	BV: NR	BV: 8	BV: NR	BV: 18	BV: NR	BV: 11	
Chiang et al., 2014 [19]	MV: NR	MV: NR	MV: 566	MV: 194	MV: 184	MV: 333	MV: 187	MV: 57	MV: 49	MV: 320	MV: 34	MV: 40	MV: NR	
	BV: NR	BV: NR	BV: 569	BV: 197	BV: 167	BV: 332	BV: 182	BV: 59	BV: 47	BV: 313	BV: 35	BV: 40	BV: NR	
Rodríguez-Caulo et al., 2021 [20]	MV: NR	MV: 929	MV: 1181	MV: 450	MV: 259	MV: NR	MV: 86	MV: 90	MV: 168	MV: NR	MV: 145	MV: NR	MV: 116	
	BV: NR	BV: 468	BV: 568	BV: 216	BV: 138	BV: NR	BV: 43	BV: 48	BV: 92	BV: NR	BV: 79	BV: NR	BV: 62	
Glaser et al., 2015 [21]	MV: NR	MV: 101	MV: 242	MV: 146	MV: 69	MV: NR	MV: 89	MV: 60	MV: NR	MV: 141	MV: 42	MV: 61	MV: 60	
	BV: NR	BV: 95	BV: 236	BV: 147	BV: 74	BV: NR	BV: 108	BV: 70	BV: NR	BV: 165	BV: 37	BV: 57	BV: 68	
Attia et al., 2021 [22]	MV: 268	MV: NR	MV: 293	MV: 69	MV: 48	MV: NR	MV: 27	MV: 34	MV: NR	MV: NR	MV: 16	MV: NR	MV: 37	
	BV: 272	BV: NR	BV: 300	BV: 69	BV: 58	BV: NR	BV: 30	BV: 31	BV: NR	BV: NR	BV: 13	BV: NR	BV: 31	
Vogt et al., 2022 [23]	MV: NR	MV: NR	MV: 47	MV: 123	MV: 34	MV: 81	MV: 30	MV: 1	MV: NR	MV: NR	MV: 31	MV: NR	MV: 24	
	BV: NR	BV: NR	BV: 51	BV: 122	BV: 33	BV: 88	BV: 30	BV: 4	BV: NR	BV: NR	BV: 19	BV: NR	BV: 25	
Kim et al., 2022 [24]	MV: 194	MV: NR	MV: 467	MV: 357	MV: 73	MV: 62	MV: NR	MV: 28	MV: 65	MV: 122	MV: 2	MV: 71	MV: 0	
	BV: 195	BV: NR	BV: 467	BV: 359	BV: 75	BV: 64	BV: NR	BV: 29	BV: 69	BV: 121	BV: 5	BV: 76	BV: 0	
Hu et al., 2021 [25]	MV: NR	MV: NR	MV: 75	MV: 79	MV: 51	MV: NR	MV: 18	MV: NR	MV: 17	MV: NR	MV: 10	MV: NR	MV: NR	
	BV: NR	BV: NR	BV: 78	BV: 86	BV: 56	BV: NR	BV: 15	BV: NR	BV: 24	BV: NR	BV: 9	BV: NR	BV: NR	
Huckaby et al., 2020 [26]	MV: NR	MV: NR	MV: 153	MV: 64	MV: 40	MV: NR	MV: NR	MV: 26	MV: NR	MV: 57	MV: 18	MV: NR	MV: 31	
	BV: NR	BV: NR	BV: 154	BV: 64	BV: 41	BV: NR	BV: NR	BV: 21	BV: NR	BV: 53	BV: 25	BV: NR	BV: 30	
Iribarne et al., 2019 [27]	MV: NR	MV: NR	MV: NR	MV: 19	MV: 15	MV: 0	MV: 9	MV: NR	MV: NR	MV: 35	MV: 12	MV: NR	MV: 0	
	BV: NR	BV: NR	BV: NR	BV: 20	BV: 14	BV: 1	BV: 8	BV: NR	BV: NR	BV: 31	BV: 13	BV: NR	BV: 1	
Alex et al., 2017 [28]	MV: 13	MV: 45	MV: 64	MV: 28	MV: 8	MV: NR	MV: 6	MV: NR	MV: NR	MV: 17	MV: 10	MV: NR	MV: 14	
	BV: 24	BV: 64	BV: 71	BV: 25	BV: 7	BV: NR	BV: 6	BV: NR	BV: NR	BV: 11	BV: 11	BV: NR	BV: 19	
Sakamoto et al., 2016 [29]	MV: NR	MV: NR	MV: NR	MV: 2	MV: NR	MV: NR	MV: NR	MV: NR	MV: 2	MV: NR	MV: 2	MV: NR	MV: NR	
	BV: NR	BV: NR	BV: NR	BV: 3	BV: NR	BV: NR	BV: NR	BV: NR	BV: 4	BV: NR	BV: 2	BV: NR	BV: NR	
Roumieh et al., 2014 [30]	MV: NR	MV: NR	MV: NR	MV: NR	MV: NR	MV: NR	MV: NR	MV: NR	MV: NR	MV: NR	MV: NR	MV: NR	MV: NR	
	BV: NR	BV: NR	BV: NR	BV: NR	BV: NR	BV: NR	BV: NR	BV: NR	BV: NR	BV: NR	BV: NR	BV: NR	BV: NR	

**Table 6:** Clinical characteristics (valvular etiology, surgical intervention, intraoperative characteristics) of each included study BV: Bioprosthetic valve; CABG: Coronary artery bypass graph; MV: Mechanical valve; n: Number of participants; NR: Not reported; PCI: Percutaneous coronary intervention

Study	Valvular etiology					Surgical intervention		Intraoperative characteristics	
	Degenerative (n)	Rheumatic (n)	Aortic insufficiency (n)	Aortic stenosis (n)	Others (n)	Elective (n)	Urgent (n)	Concomitant CABG (n)	PCI (n)
Stassano et al., 2009 [18]	MV: NR	MV: NR	MV: NR	MV: NR	MV: NR	MV: NR	MV: 11	MV: 36	MV: NR
	BV: NR	BV: NR	BV: NR	BV: NR	BV: NR	BV: NR	BV: 12	BV: 43	BV: NR
Chiang et al., 2014 [19]	MV: NR	MV: NR	MV: NR	MV: NR	MV: NR	MV: NR	MV: 364	MV: 43	MV: 21
	BV: NR	BV: NR	BV: NR	BV: NR	BV: NR	BV: NR	BV: 351	BV: 41	BV: 19
Rodríguez-Caulo et al., 2021 [20]	MV: NR	MV: NR	MV: NR	MV: NR	MV: NR	MV: NR	MV: NR	MV: NR	MV: NR
	BV: NR	BV: NR	BV: NR	BV: NR	BV: NR	BV: NR	BV: NR	BV: NR	BV: NR
Glaser et al., 2015 [21]	MV: NR	MV: NR	MV: NR	MV: NR	MV: NR	MV: NR	MV: 21	MV: NR	MV: 29
	BV: NR	BV: NR	BV: NR	BV: NR	BV: NR	BV: NR	BV: 21	BV: NR	BV: 18
Attia et al., 2021 [22]	MV: NR	MV: NR	MV: NR	MV: NR	MV: 247	MV: NR	MV: 1	MV: NR	MV: NR
	BV: NR	BV: NR	BV: NR	BV: NR	BV: 275	BV: NR	BV: 0	BV: NR	BV: NR
Vogt et al., 2022 [23]	MV: NR	MV: NR	MV: NR	MV: NR	MV: NR	MV: 522	MV: 88	MV: NR	MV: 28
	BV: NR	BV: NR	BV: NR	BV: NR	BV: NR	BV: 511	BV: 99	BV: NR	BV: 34
Kim et al., 2022 [24]	MV: NR	MV: NR	MV: 558	MV: 1224	MV: NR	MV: NR	MV: NR	MV: NR	MV: NR
	BV: NR	BV: NR	BV: 540	BV: 1203	BV: NR	BV: NR	BV: NR	BV: NR	BV: NR
Hu et al., 2021 [25]	MV: 150	MV: 157	MV: NR	MV: 114	MV: NR	MV: NR	MV: 11	MV: 346	MV: NR
	BV: 158	BV: 146	BV: NR	BV: 103	BV: NR	BV: NR	BV: 12	BV: 346	BV: NR
Huckaby et al., 2020 [26]	MV: 39	MV: 5	MV: 165	MV: 179	MV: 77	MV: 159	MV: 37	MV: 11	MV: 17
	BV: 40	BV: 6	BV: 160	BV: 179	BV: 68	BV: 161	BV: 35	BV: 11	BV: 15
Iribarne et al., 2019 [27]	MV: NR	MV: NR	MV: NR	MV: NR	MV: NR	MV: 78	MV: 22	MV: 2	MV: 5
	BV: NR	BV: NR	BV: NR	BV: NR	BV: NR	BV: 79	BV: 21	BV: 2	BV: 5
Alex et al., 2017 [28]	MV: NR	MV: NR	MV: NR	MV: 90	MV: NR	MV: 98	MV: 20	MV: 41	MV: NR
	BV: NR	BV: NR	BV: NR	BV: 95	BV: NR	BV: 98	BV: 20	BV: 42	BV: NR
Sakamoto et al., 2016 [29]	MV: NR	MV: NR	MV: NR	MV: NR	MV: NR	MV: NR	MV: NR	MV: 3	MV: NR
	BV: NR	BV: NR	BV: NR	BV: NR	BV: NR	BV: NR	BV: NR	BV: 3	BV: NR
Roumieh et al., 2014 [30]	MV: NR	MV: NR	MV: NR	MV: 28	MV: 3	MV: NR	MV: NR	MV: 0	MV: NR
	BV: NR	BV: NR	BV: NR	BV: 20	BV: 3	BV: NR	BV: NR	BV: 0	BV: NR

Risk of Bias

Studies of good quality were defined as having a rating of 6 and above. All studies included were judged to be of fair or good quality as defined by the standards of the Agency for Healthcare Research and Quality [31]. The patients appeared to represent the whole experience of the investigator. The exposure and outcome were adequately ascertained, and the lengths of follow-up were adequate. However, only three studies reported on adequacy of follow-up [23,24,29]. Results of the quality assessment of all included studies are detailed in Table 7.

**Table 7:** Newcastle-Ottawa Quality Assessment scale † Stassano et al. (2009) was evaluated as an RCT. Scoring explanation for each category (a maximum of one star can awarded for each category with the exception of two stars in the comparability category): - Representativeness of the exposed cohort: A* - truly representative; B* - somewhat representative - Selection of the non-exposed cohort: A* - drawn from the same community as the exposed cohort; N/A - not applicable - Ascertainment of exposure: A* - secure records - Demonstration that outcome of interest was not present at the start of the study: A* - yes - Comparability: * study controls for one important factor, ** study controls for two important factors - Assessment of outcome: B* - record linkage - Follow-up: A* - adequate follow-up for outcomes to occur - Adequacy of follow-up of cohorts: B* - subjects lost to follow-up unlikely to introduce bias or description provided of those lost; C - no description provided; D - no statement Good quality: 3 or 4 stars (*) in the selection domain AND 1 or 2 stars in the comparability domain AND 2 or 3 stars in the outcome domain; Fair quality: 2 stars in the selection domain AND 1 or 2 stars in the comparability domain AND 2 or 3 stars in the outcome/exposure domain; Poor quality: 0 or 1 star in selection domain OR 0 stars in the comparability domain OR 0 or 1 stars in the outcome/exposure domain.

Study	Selection				Comparability		Outcomes/exposure			Quality score
	Representativeness of the exposed cohort	Selection of non-exposed cohort	Ascertainment of exposure	Demonstration that outcome of interest was not present at the start of the study	Study controls for (ethnicity)	Study controls for any additional factor (follow-up time, age, urgency, comorbidities)	Assessment of outcome	Was follow-up long enough for outcomes to occur?	Adequacy of follow-up of cohorts	
Stassano et al., 2009 [18] †	A*	A*	A*	B	-	*	C	A*	A*	6
Chiang et al., 2014 [19]	A*	A*	A*	A*	*	*	D	A*	D	7
Rodriguez-Caulo et al., 2021 [20]	A*	A*	A*	A*	-	*	B*	A*	D	7
Glaser et al., 2015 [21]	A*	A*	A*	A*	-	*	B*	A*	D	7
Attia et al., 2021 [22]	A*	A*	A*	B	-	*	B*	A*	D	7
Vogt et al., 2022 [23]	A*	A*	A*	A*	-	*	B*	A*	A*	8
Kim et al., 2022 [24]	A*	A*	A*	A*	-	*	C	A*	A*	7
Hu et al., 2021 [25]	A*	A*	A*	A*	-	*	B*	A*	D	7
Huckaby et al., 2020 [26]	A*	A*	A*	A*	*	*	D	A*	D	7
Iribarne et al., 2019 [27]	A*	A*	A*	A*	-	*	B*	A*	D	7
Alex et al., 2017 [28]	A*	A*	A*	A*	-	*	C	A*	D	6
Sakamoto et al., 2016 [29]	A*	A*	A*	A*	-	*	B*	A*	A*	8
Roumieh et al., 2014 [30]	A*	A*	A*	B	-	*	B*	A*	D	6

Long-Term Survival

**Figure 2:**
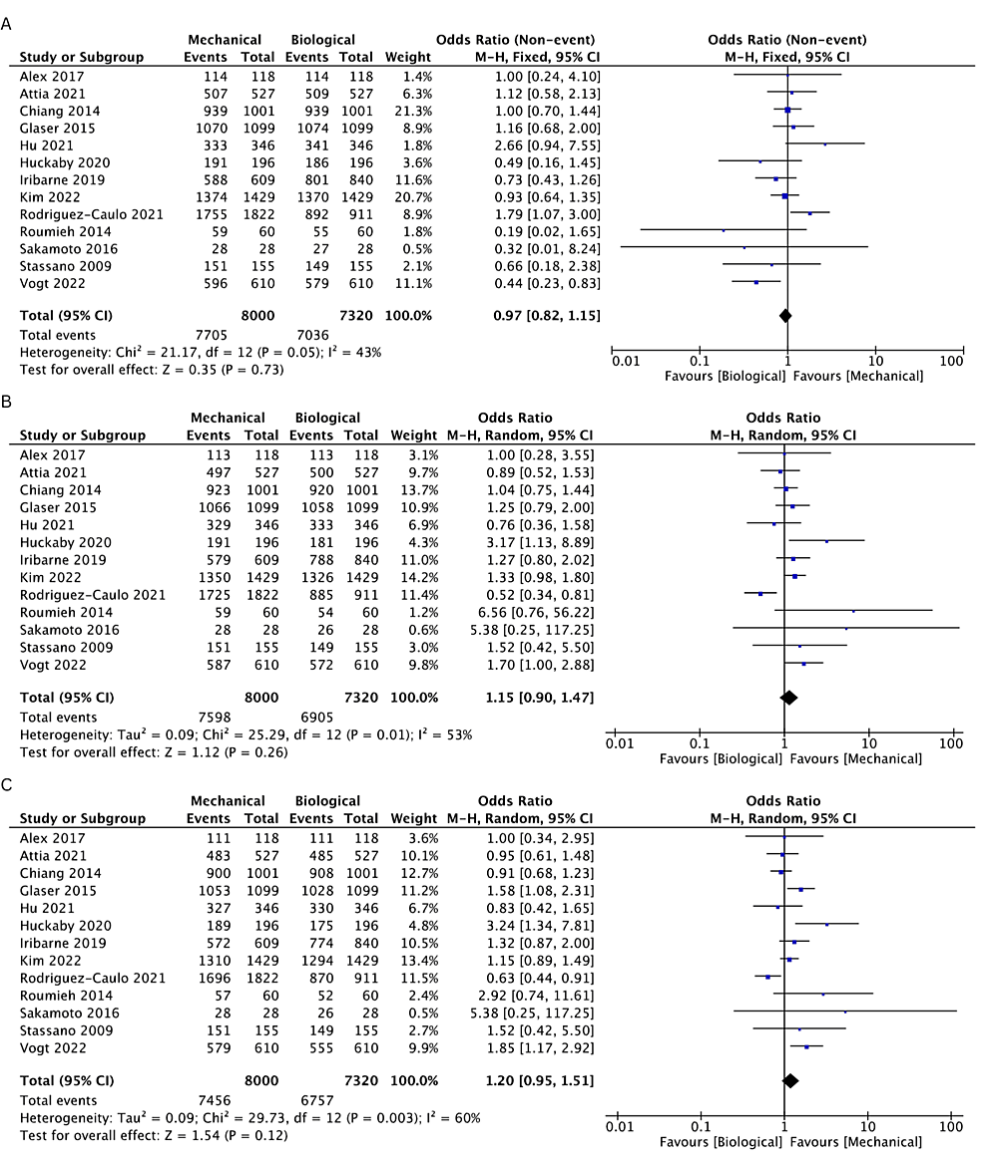
A pooled estimate of the overall survival rate between mechanical and biological valve replacement at 1 (A), 2 (B), and 3 (C) years Reference: Alex [28], Attia [22], Chiang [19], Glaser [21], Hu [25], Huckaby [26], Iribarne [27], Kim [24], Rodriguez-Caulo [20], Roumieh [30], Sakamoto [29], Stassano [18], Vogt [23]

**Figure 3:**
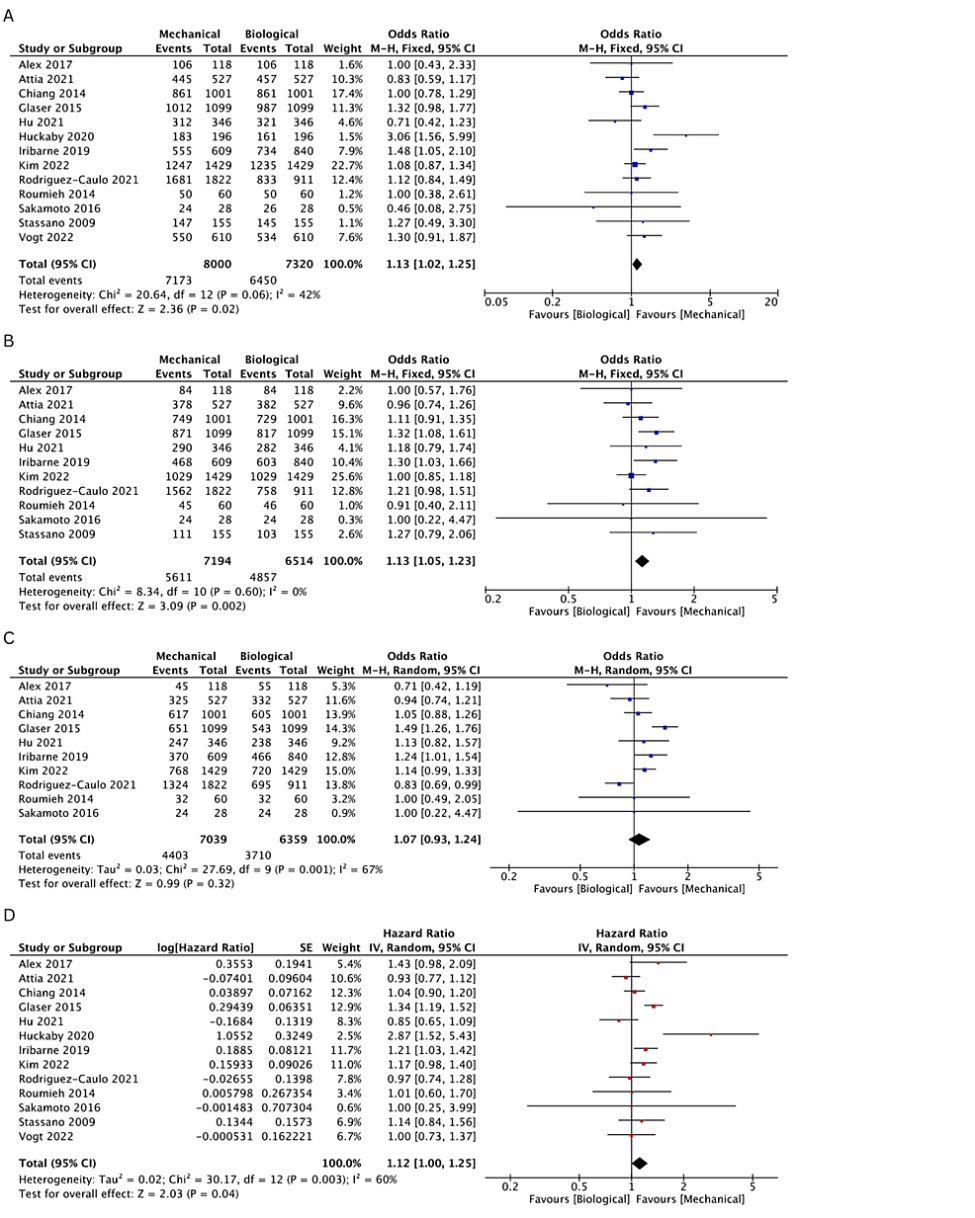
A pooled estimate of the overall survival rate between mechanical and biological valve replacement at 5 (A), 10 (B), and 15 (C) years with the hazard ratio (D) Reference: Alex [28], Attia [22], Chiang [19], Glaser [21], Hu [25], Huckaby [26], Iribarne [27], Kim [24], Rodriguez-Caulo [20], Roumieh [30], Sakamoto [29], Stassano [18], Vogt [23]

All included studies reported survival rates in both MV and BV groups [18-30]. The overall HR of each included study was pooled to determine the overall long-term survival. The MV cohort depicted a more favorable HR compared to the BV cohort (HR: 1.12, 95% CI: 1.00-1.25, I^2^ = 60%). The pooled estimate of survival rates at 1 year (OR: 0.97, 95% CI: 0.82-1.15, I^2^ = 43%), 2 years (OR: 1.15, 95% CI: 0.90-1.47, I^2^ = 53%) 3 years (OR: 1.20, 95% CI: 0.95-1.51, I^2^ = 60%) and 15 years (OR: 1.07, 95% CI: 0.93-1.24, I^2^ = 67%) were found comparable between the MV and BV groups. However, the MV cohort reported higher rates of survival compared to the BV cohort at 5 (OR: 1.13, 95% CI: 1.02-1.25, I^2^ = 42%) and 10 years (OR: 1.13, 95% CI: 1.05-1.23, I^2^ = 0%). The survival rates for the studies included are comprehensively illustrated in Figures 2A-2C and Figures 3A-3D.

Incidence of Stroke

**Figure 4:**
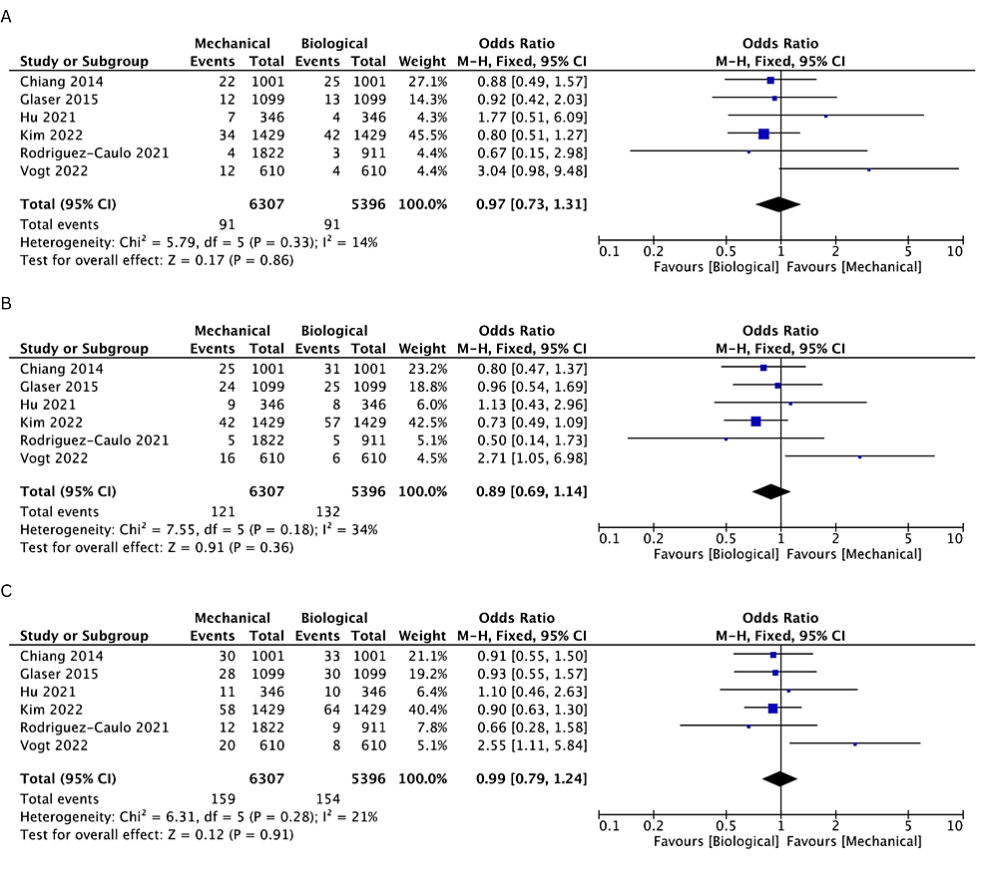
A pooled estimate of stroke incidence between mechanical and biological valve replacement at 1 (A), 2 (B), and 3 (C) years Reference: Chiang [19], Glaser [21], Hu [25], Kim [24], Rodriguez-Caulo [20], Vogt [23]

**Figure 5:**
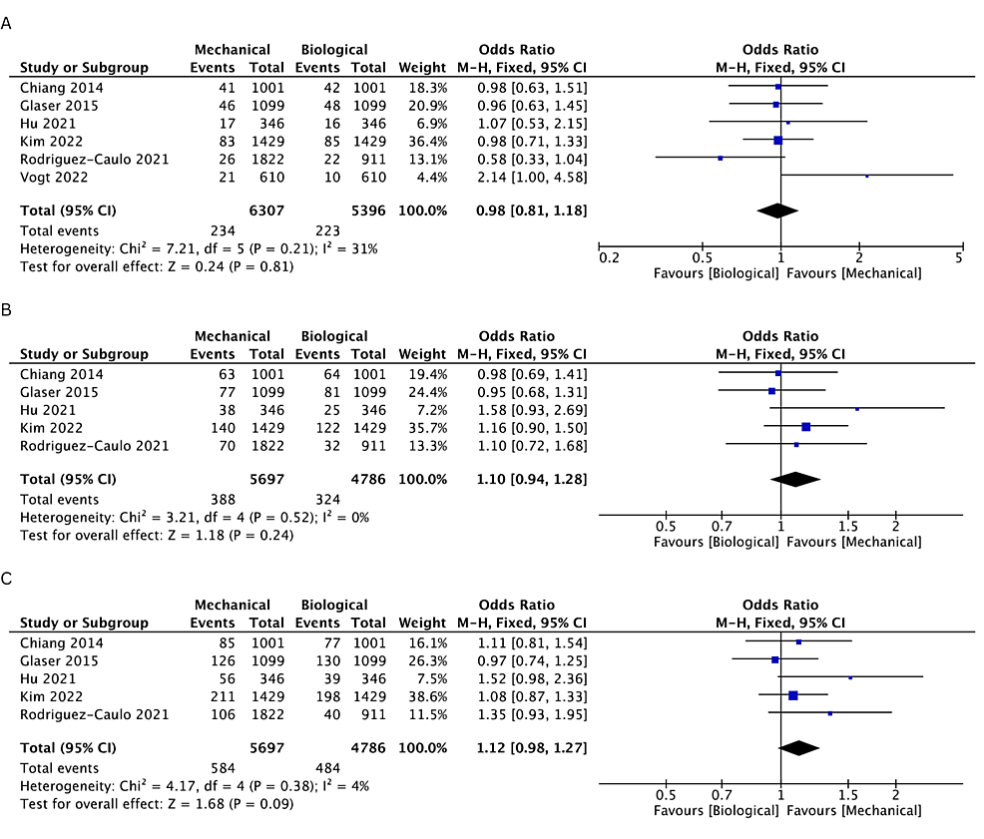
A pooled estimate of stroke incidence between mechanical and biological valve replacement at 5 (A), 10 (B), and 15 (C) years Reference: Chiang [19], Glaser [21], Hu [25], Kim [24], Rodriguez-Caulo [20], Vogt [23]

The incidence of stroke was evaluated in six studies [19-21,23-25]. Both groups were found comparable for incidence of stroke events at 1 (OR: 0.97, 95% CI: 0.73-1.31, I^2^ = 14%), 2 (OR: 0.89, 95% CI: 0.69-1.14, I^2 ^= 34%), 3 (OR: 0.99, 95% CI: 0.79-1.24, I^2^ = 21%), 5 (OR: 0.98, 95% CI: 0.81-1.18, I^2^ = 31%), 10 (OR: 1.1, 95% CI: 0.94-1.28, I^2^ = 0%), and 15 years (OR: 1.12, 95% CI: 0.98-1.27, I^2^ = 4%). The rates of incidence of stroke events are comprehensively illustrated in Figures 4A-4C and Figures 5A-5C.

Incidence of Bleeding

**Figure 6:**
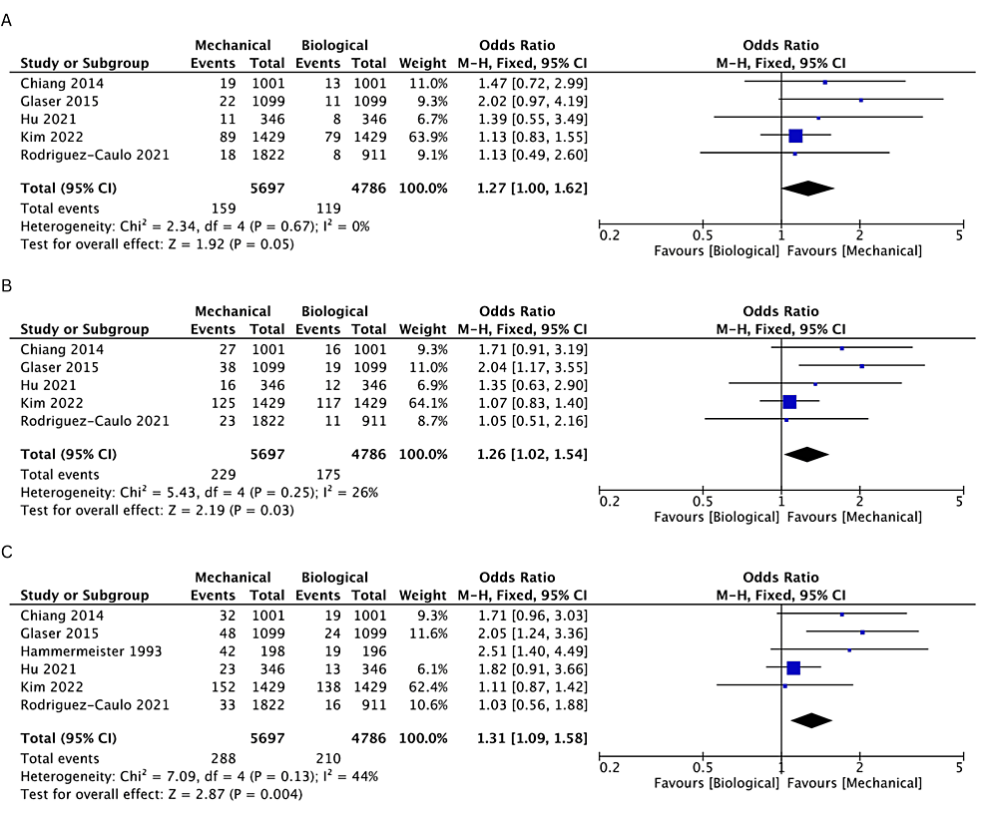
A pooled estimate of bleeding incidence between mechanical and biological valve replacement at 1 (A), 2 (B), and 3 (C) years Reference: Chiang [19], Glaser [21], Hu [25], Kim [24], Rodriguez-Caulo [20]

**Figure 7:**
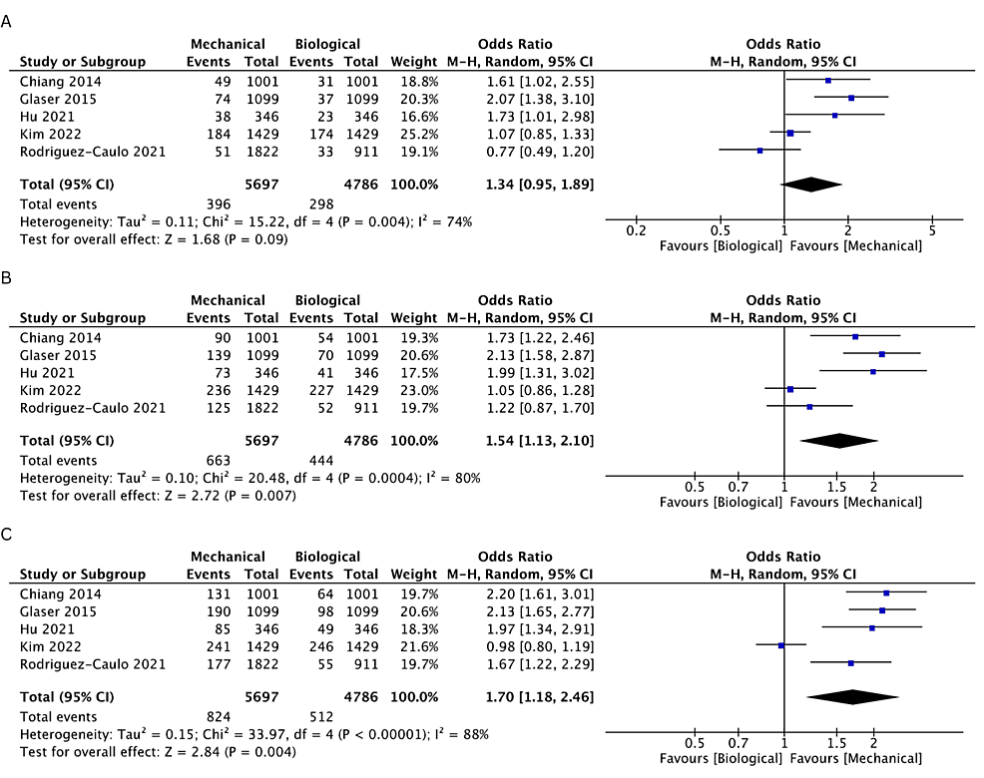
A pooled estimate of bleeding incidence between mechanical and biological valve replacement at 5 (A), 10 (B), and 15 (C) years Reference: Chiang [19], Glaser [21], Hu [25], Kim [24], Rodriguez-Caulo [20]

Bleeding incidence was reported in five studies [19-21,24,25]. There was a higher incidence of bleeding in the MV cohort at 1 (OR: 1.27, 95% CI: 1.00-1.62, I^2^ = 0%), 2 (OR: 1.26, 95% CI: 1.02-1.54, I^2^ = 26%), 3 (OR: 1.31, 95% CI: 1.09-1.58, I^2^ = 44%), 10 (OR: 1.54, 95% CI: 1.13-2.1, I^2^ = 80%), and 15 years (OR: 1.7, 95% CI: 1.18-2.46, I^2^ = 88%), but results were comparable between both groups at 5 years (OR: 1.34, 95% CI: 0.95-1.89, I^2^ = 74%). The rates of freedom from bleeding events are comprehensively illustrated in Figures 6A-6C and Figures 7A-7C.

Incidence of Reoperation

**Figure 8:**
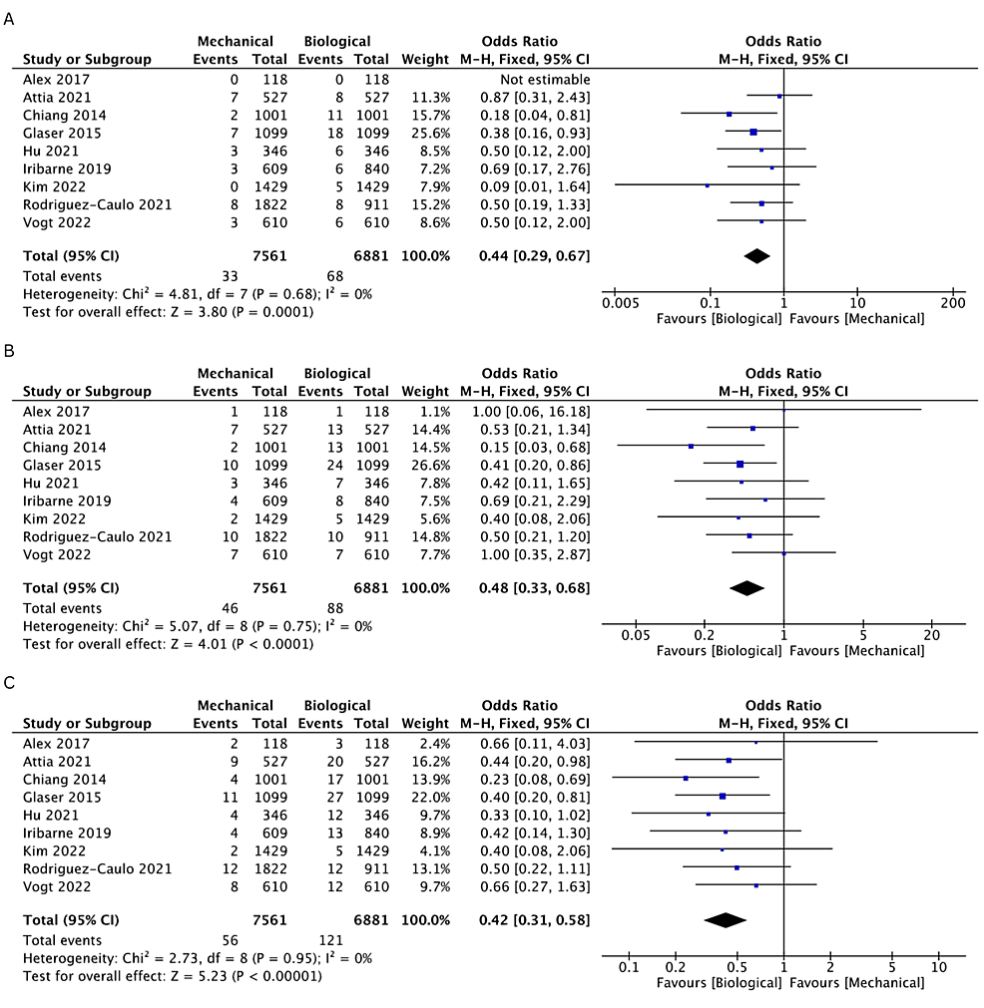
A pooled estimate of reoperation incidence between mechanical and biological valve replacement at 1 (A), 2 (B), and 3 (C) years Reference: Alex [28], Attia [22], Chiang [19], Glaser [21], Hu [25], Iribarne [27], Kim [24], Rodriguez-Caulo [20], Vogt [23]

**Figure 9:**
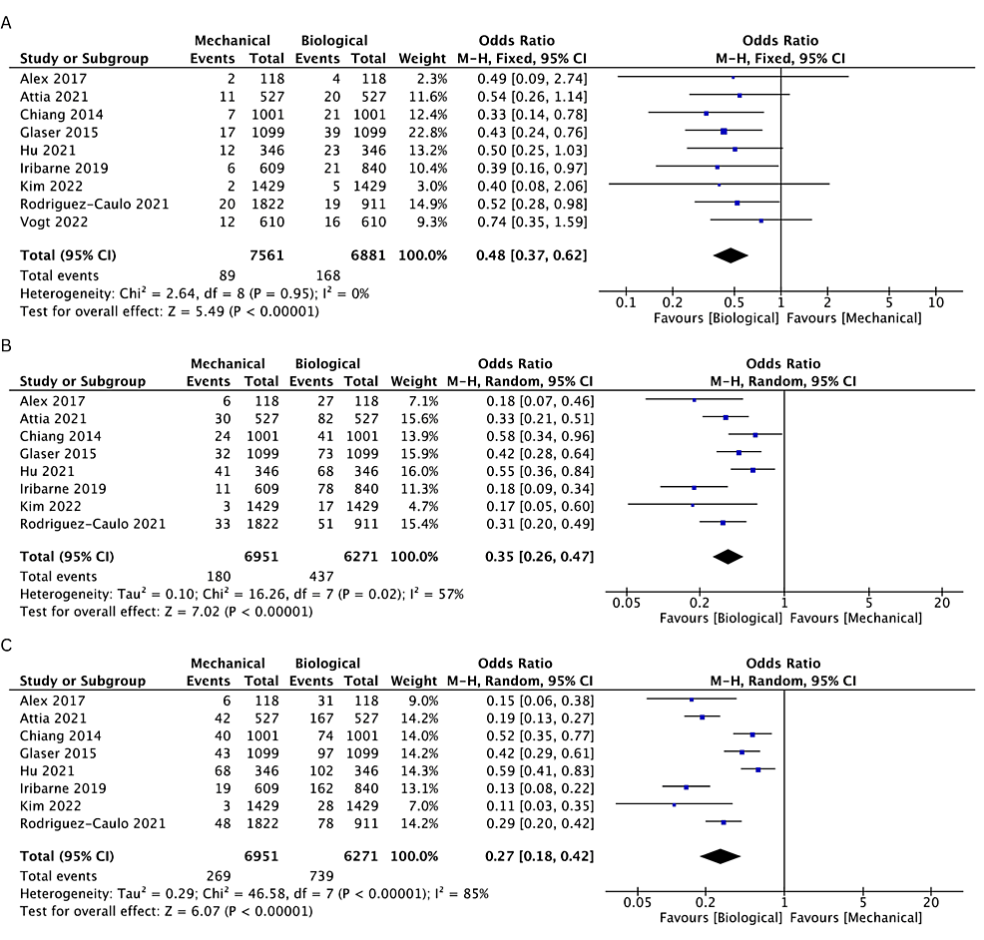
A pooled estimate of reoperation incidence between mechanical and biological valve replacement at 5 (A), 10 (B), and 15 (C) years Reference: Alex [28], Attia [22], Chiang [19], Glaser [21], Hu [25], Iribarne [27], Kim [24], Rodriguez-Caulo [20], Vogt [23]

Nine studies [19-25,27,28] reported the incidence of reoperation events. The BV cohort was found to have a higher incidence of reoperation rates compared to the MV cohort at 1 (OR: 0.44, 95% CI: 0.29-0.67, I^2^ = 0%), 2 (OR: 0.48, 95% CI: 0.33-0.68, I^2^ = 0%), 3 (OR: 0.42, 95% CI: 0.31-0.58, I^2^ = 0%), 5 (OR: 0.48, 95% CI: 0.37-0.62, I^2^ = 0%), 10 (OR: 0.35, 95% CI: 0.26-0.47, I^2^ = 57%), and 15 years (OR: 0.27, 95% CI: 0.18-0.42, I^2^ = 85%). The rates of incidence of reoperation are comprehensively illustrated in Figures 8A-8C and Figures 9A-9C.

A summary of the meta-analysis results can be found in Table 8.

**Table 8:** Summary of the meta-analysis results BV: Bioprosthetic valve; CI: Confidence interval; MV: Mechanical valve; N/A: Not applicable

	Odds ratio	95% CI	I^2^	MV proportion	BV proportion	Number of studies included
Survival rates						
1 year	0.97	0.82-1.15	43%	7705/8000	7036/7320	13
2 years	1.15	0.90-1.47	53%	7598/8000	6905/7320	13
3 years	1.2	0.95-1.51	60%	7456/8000	6757/7320	13
5 years	1.13	1.02-1.25	42%	7173/8000	6450/7320	13
10 years	1.13	1.05-1.23	0%	5611/7194	4857/6514	11
15 years	1.07	0.93-1.24	67%	4403/7039	3710/6359	10
Overall hazard ratio	1.12	1.00-1.25	60%	N/A	N/A	13
Incidence of stroke						
1 year	0.97	0.73-1.31	14%	91/6307	91/5396	6
2 years	0.89	0.69-1.14	34%	121/6307	132/5396	6
3 years	0.99	0.79-1.24	21%	159/6307	154/5396	6
5 years	0.98	0.81-1.18	31%	234/6307	223/5396	6
10 years	1.1	0.94-1.28	0%	388/5697	324/4786	5
15 years	1.12	0.98-1.27	4%	584/5697	484/4786	5
Incidence of bleeding						
1 year	1.27	1.00-1.62	0%	159/5697	119/4786	5
2 years	1.26	1.02-1.54	26%	229/5697	175/4786	5
3 years	1.31	1.09-1.58	44%	288/5697	210/4786	5
5 years	1.34	0.95-1.89	74%	396/5697	298/4786	5
10 years	1.54	1.13-2.10	80%	663/5697	444/4786	5
15 years	1.7	1.18-2.46	88%	824/5697	512/4786	5
Incidence of reoperation						
1 year	0.44	0.29-0.67	0%	33/7561	68/6881	9
2 years	0.48	0.33-0.68	0%	46/7561	88/6881	9
3 years	0.42	0.31-0.58	0%	56/7561	121/6881	9
5 years	0.48	0.37-0.62	0%	89/7561	168/6881	9
10 years	0.35	0.26-0.47	57%	180/6951	437/6271	8
15 years	0.27	0.18-0.42	85%	269/6951	739/6271	8

Discussion

Guidelines on aortic mechanical valve (MV) and BV replacement between the ages of 50 and 70 years remain without definitive recommendations. This may be in part due to deficiencies in updated and extended understanding regarding survival rates and freedom from stroke, bleeding, and reoperation. This meta-analysis is the first to report on long-term clinical outcomes over a time period of 15 years with data presented for 1, 2, 3, 5, 10, and 15 years postoperatively. It was found that the MV cohort demonstrated favorable survival at 5 and 10 years postoperatively, whereas the BV cohort demonstrated favorable results for postoperative risk of bleeding. To our knowledge, this meta-analysis is the first to offer a time-specific analysis of survival. This in turn allows a deeper understanding of the progression of results for the MV and BV cohorts to better equip patients and physicians in determining the choice of valve type.

The ACC has guidelines regarding the choice of valve replacement, yet no consensus has been reached for patients in the age group of 50-65 years [32]. With the addition of a time-specific analysis of survival, our results were able to show an increased rate of survival in the MV cohort at 5 and 10 years postoperatively as well as for hazard of mortality. Similarly, Badhwar et al. (2012) reported that the survival benefit in the MV cohort peaks at 7.5 years postoperatively when propensity-matched with the BV cohort [33]. A previous meta-analysis completed a meta-regression analysis after finding similar results and found a correlation between increasing publication dates and decreasing differences in overall survival between the two cohorts [11]. The reasoning for such differences in survival has yet to be discovered; however, theories suggest that increased structural valve degeneration and corresponding hemodynamic changes in the BV cohort may contribute to decreased overall survival rates [11]. With the increasing rates of BV replacements over MVs, it is imperative to conduct more trials investigating survival differences for these cohorts, as the results may lead to changes in guidelines showing favor towards MVs.

The current ACC guidelines report the use of long-term anticoagulants for the prevention of strokes in MV replacements, which might potentially lead to a preference for the bioprosthetic route [32]. This meta-analysis found no difference in overall stroke rates for the MV and BV cohorts, and this is in line with the results of a 2019 meta-analysis, which, however, included only four studies in the evaluation of this outcome [34]. Keeping in mind that the majority of postoperative strokes are embolic in nature, it should be noted that valve type can lead to a difference in pathophysiology. The known thrombogenicity of MV is one of the main causes of stroke in this cohort and the reason for necessary long-term anticoagulation [35]. In contrast, the cause of stroke in the BV cohort is largely debated but theorized to be due to undetected atrial fibrillation and lack of anticoagulation [36]. Consequently, there is a reported increase in the incidence of atrial fibrillation at the median age of 71.8 years necessitating an increased need for long-term anticoagulation in these patients as well [35]. The thromboembolic risk for those with postoperative development of atrial fibrillation and long-term use of anticoagulants is similar to those with atrial fibrillation alone [37]. This counterbalance of causes in each cohort may contribute to the results found in this meta-analysis [36]. Alternatively, the correct usage and monitoring of long-term anticoagulants in the MV cohort may have also contributed to this result by properly preventing postoperative stroke. More trials are needed to further elucidate the relationship between valve type, causes, and incidence of stroke.

Furthermore, the use of long-term anticoagulation is largely associated with increased rates of bleeding [33]. This meta-analysis showed lower incidents of bleeding in the BV cohort in comparison to the MV cohort. A 2022 meta-analysis found similar results of lower odds for major bleeding events in the BV cohort. However, their follow-up times were dependent on the mean follow-up times of each included study and therefore only investigated bleeding as an overall late outcome. As vitamin K antagonists are the primary choice of anticoagulation, this comes with the debate surrounding international normalized ratio (INR) monitoring. The use of self-testing in Germany allowed for greater time in therapeutic range (TTR) and therefore lowered the risk of thromboembolic events (HR: 0.51, 95% CI: 0.31-0.85) [38]. In addition, they reported a decrease in severe hemorrhagic complications and noted higher therapeutic compliance within the self-monitoring cohort, which may contribute to the results found [38]. Contradictory results were shown in a more recent study that reported no difference regarding TTR for in-home monitoring and in-clinic monitoring, which was attributed to a difference in INR goals as well as a difference in the self-management dosing regimen [39]. Current ACC guidelines recommend an INR goal of 2-3 for those at low risk of thromboembolic events and 2.5-3.5 for those at high risk of thromboembolic events [32]. However, INR monitoring slightly differs between regions and publication dates as INR goals are continually changing with updated research. With this, more trials are needed to improve the methods of monitoring INR to optimize the incidence of stroke and freedom from bleeding in the MV cohort.

With the need for long-term use of anticoagulants in the MV cohort and corresponding increased risk of bleeding, there has been an increased number of patients and physicians choosing BV, therefore increasing rates of reoperation. This meta-analysis found a decreased need for reoperation in the MV cohort for all reported years, as well as a decreased hazard of reoperation. Similar results were found in the meta-analyses completed in 2019 (incidence rate ratio (IRR): 0.46, 95% CI: 0.35-0.60) and 2022 (OR: 1.75, 95% CI: 1.41-2.16) [11,34]. The most common causes of valve reoperation include infective endocarditis and valve deterioration in the form of cusp tear, perforation, stretching, and thickening. Some found similar rates of infective endocarditis in both cohorts and more frequent incidences of deterioration in the BV cohort [35]. Conversely, a nationwide study completed in Denmark found a higher risk of infective endocarditis in the BV cohort compared to MV [40]. Along with the high mortality rate surrounding infective endocarditis, reoperation is often coupled with patient factors such as increased patient comorbidities, surgical scarring, and sternal re-entries [41,42], which make it a high-risk operation. Therefore, the need to decrease reoperations is imperative. With a high incidence of reoperation in the BV cohort, many trials have looked at the safety and efficacy of surgical redo-AVR (rAVR) and the method of valve-in-valve transcatheter AVR (ViV), with the surgical option standing as the gold standard. A recent meta-analysis comparing ViV with rAVR reported better short-term survival in the ViV cohort, yet no difference was found between the cohorts at mid-term follow-up [42]. Multiple factors may play a role in this result, such as greater short-term risk with invasive surgeries, greater hemodynamic instability in the ViV cohort, or more patients within the ViV being considered high risk in regards to their age and comorbidities [42]. With increased incidences of BV replacements, there is a projected increased need for redo-operations in the future and therefore more trials must be conducted to further elucidate the safety and efficacy of the ViV method of replacement.

As with all meta-analyses, there are limitations to this study. First, propensity score-matched data was the predominant source for analysis. The use of retrospective analysis introduces unknown confounding and limits the patient sample size. This otherwise can be eliminated using prospective randomized controlled trials. However, assumptions of reduced confounding within PSM and RCT must be made to yield data synthesis and reduce study bias. Nonetheless, the potential risk of unknown confounding must be further acknowledged in longitudinal studies. Second, our methodology utilized a reconstructed database from Kaplan Meier curves. The quality and accuracy of data extraction using a web plot digitizer were dependent on image quality. Although a comparison between reconstructed data and HR/IRR was performed for reliability, discrepancies exist when compared to the original data. Furthermore, data reconstruction of secondary outcomes required conversion of data sets into freedom from event and standardization of all data sets to compare mechanical vs BVs. Third, current guidelines vary for age groups of <50, 50-70, and >70 years. However, the majority of articles did not include exact age groups between 50 and 70 years of age. Instead, age ranges of 55-65 and 60-70 that were provided from original data and were cumulatively recorded further introduced possible bias and confounding and limited subgroup analysis. Fourth, another limitation encountered was a lack of perioperative comorbidities across all articles. Baseline risk scores for respective conditions, like diabetes or existing coagulopathies, may impact the type of prosthesis received despite age group. Last, the lack of clarification for the exact types of bioprosthetic and mechanical valves used may contribute to survival expectancy.

## Conclusions

The choice of AVR type is dependent on the complexity of patient-dependent factors. Despite the above, overarching guidelines exist in accordance with patient age. This meta-analysis has shown an increased hazard of survival and freedom from reoperation with MV replacements compared to BV. MVs are thus a suitable choice for patients aged 50-70 years with no contraindications for long-term anticoagulation use. This meta-analysis aims to serve as a guide for future updates in the ACC guidelines regarding the choice of valve replacement in the age group of 50-70. Last, this meta-analysis hopes to provide a foundation for future trials with the goal of optimizing survival and freedom from stroke, bleeding, and reoperation.
